# Children’s Environmental Health Faculty Champions Initiative: A Successful Model for Integrating Environmental Health into Pediatric Health Care

**DOI:** 10.1289/ehp.0800203

**Published:** 2008-12-05

**Authors:** Bonnie Rogers, Leyla Erk McCurdy, Katie Slavin, Kimberly Grubb, James R. Roberts

**Affiliations:** 1 School of Public Health, University of North Carolina at Chapel Hill, Chapel Hill, North Carolina, USA;; 2 National Environmental Education Foundation, Washington, DC, USA;; 3 American Nurses Association, Silver Spring, Maryland, USA;; 4 Medical University of South Carolina, Charleston, South Carolina, USA

**Keywords:** children, education, environmental health, medicine, medical schools, nursing, nursing schools, pediatrics

## Abstract

**Background:**

Pediatric medical and nursing education lack the environmental health content needed to properly prepare health care professionals to prevent, recognize, manage, and treat environmental exposure–related diseases. The need for improvements in health care professionals’ environmental health knowledge has been expressed by leading institutions. However, few studies have evaluated the effectiveness of programs that incorporate pediatric environmental health (PEH) into curricula and practice.

**Objective:**

We evaluated the effectiveness of the National Environmental Education Foundation’s (NEEF) Children’s Environmental Health Faculty Champions Initiative, which is designed to build environmental health capacity among pediatric health care professionals.

**Methods:**

Twenty-eight pediatric health care professionals participated in a train-the-trainer workshop, in which they were educated to train other health care professionals in PEH and integrate identified PEH competencies into medical and nursing practice and curricula. We evaluated the program using a workshop evaluation tool, action plan, pre- and posttests, baseline and progress assessments, and telephone interviews.

**Results:**

During the 12 months following the workshop, the faculty champions’ average pretest score of 52% was significantly elevated (*p* < 0.0001) to 65.5% on the first posttest and to 71.5% on the second posttest, showing an increase and retention of environmental health knowledge. Faculty champions trained 1,559 health care professionals in PEH, exceeding the goal of 280 health care professionals trained. Ninety percent of faculty champions reported that PEH had been integrated into the curricula at their institution.

**Conclusion:**

The initiative was highly effective in achieving its goal of building environmental health capacity among health care professionals. The faculty champions model is a successful method and can be replicated in other arenas.

Although adults and children alike are at risk for developing disease from hazardous exposures, children have special vulnerabilities in regard to their exposures and responses to the environment. In proportion to their size, they breathe in more air, drink more water, and eat more food than adults (Landrigan et al. 2002). Moreover, although children < 5 years of age make up only 12% of the world’s population, it is estimated that 43% of the total burden of disease attributable to environmental factors falls on them (Garbino 2005). In the United States alone, the total annual cost for environmentally attributable childhood disease is $54.9 billion (Landrigan et al. 2002).

Despite the economic cost and increased incidence of environmental exposure–related disease in children, pediatric medical and nursing education currently lacks the environmental health content needed to properly prepare health care professionals to prevent, recognize, manage, and treat diseases caused by environmental exposures (McCurdy et al. 2004). Most medical schools and residency programs spend a limited amount of time on environmental aspects of disease (Graber et al. 1995; Roberts and Gitterman 2003). In fact, a study of environmental medicine content in U.S. medical schools found that 75% of schools require only about 7 hours of study in environmental medicine over the 4 years of medical school (Schenk et al. 1996). Furthermore, a study of chief residents of U.S. pediatric residency programs found that fewer than half of pediatric programs routinely include pediatric environmental health (PEH) issues in their curriculum, other than lead poisoning and environmental exacerbation of asthma (Roberts and Gitterman 2003).

Several studies of health care professionals have identified the need for increased environmental health education. Kilpatrick et al. (2002) distributed a survey to pediatricians in Georgia to assess their knowledge, attitudes, and behaviors regarding patients’ environmental histories. Although more than half of the surveyed pediatricians reported having seen a patient with health issues related to environmental exposures, fewer than one in five had received training in environmental history-taking. Respondents reported low self-efficacy regarding environmental history-taking, discussing environmental exposures with parents, and finding diagnosis and treatment resources related to environmental exposures. Trasande et al. (2006a, 2006b, 2008) modeled Kilpatrick et al.’s (2002) survey assessing pediatricians attitudes, beliefs, and practices regarding pediatric environmental health, distributing the survey to pediatricians in New York, Wisconsin, and Minnesota in three separate studies. Results of each study were consistent, with pediatricians in all three states reporting a high interest in PEH issues yet feeling ill equipped to discuss common exposures and environmentally related illnesses with patients and their families. Balbus et al. (2006) found that most pediatricians and nurses felt poorly prepared to answer questions from patients on pesticides.

This need for improvements in health care professionals’ environmental health knowledge has been expressed by leading health institutions. The Institute of Medicine recommends the integration of environmental health concepts into all levels of medical and nursing education (Pope and Rall 1995; Pope et al. 1995). Furthermore, the “Health Professionals and Environmental Health Education Position Statement” (Rogers 2004), which calls on health care providers to increase their knowledge of environmental health issues and recommends the creation of faculty champions as an effective strategy for integration of environmental health knowledge into health care professionals’ education and practice, has been endorsed by > 30 leading health professional and public health institutions including the American Academy of Pediatrics, American Association of Colleges of Nursing, American College of Preventive Medicine, American Nurses Association, and American Public Health Association (NEEF 2009).

Few studies have evaluated the effectiveness of programs that incorporate PEH into curricula and practice. The purpose of this study is to evaluate the National Environmental Education Foundation’s (NEEF) Children’s Environmental Health Faculty Champions Initiative, which was designed to build health professional capacity to address children’s environmental health issues. We anticipate that the program will enhance the development of effective strategies to produce health care professionals competent in PEH.

## Methods

In this study we used a faculty champion model in which 28 faculty members from medical and nursing schools were trained in PEH at a train-the-trainer workshop. Each faculty champion committed to training 10 additional health care professionals in the 12 months after the workshop and to integrate PEH into curricula and practice at their institutions. The goals of the project were to increase the number of health care professionals able to address children’s environmental health issues in their practice, to integrate PEH into medical and nursing curricula, and to impact institutional decision making regarding the integration of PEH concepts into protocols and procedures. The project aimed to make the faculty champions proficient in five competency areas: taking PEH histories; making referrals for preventive and curative interventions for possible environmental health hazards; being involved with community groups/organizations regarding risk communication; identifying resources used to address pediatric environmental hazards; and reporting incidents for regulatory requirements.

Because this evaluation research study was conducted in a real-world setting, we used a preexperimental mixed methods design. The study had several components, including the delivery of a Children’s Environmental Health Faculty Champions train-the-trainer workshop; the completion of pre- and posttests to measure sustained knowledge acquisition; the training of additional faculty members, residents, students, and clinicians by the faculty champions; participation by the faculty champions in baseline and ongoing self-assessments of integration of PEH content into medical and nursing school curricula and practice; influences on institutional changes; and competency achievement.

### Participants

A planning committee was established to provide guidance in recruiting faculty to participate in the Faculty Champions Initiative, to assist with developing the workshop curriculum and agenda, and to support ongoing outreach efforts. The committee consisted of medical and nursing faculty members with expertise in PEH as well as representatives from the Ambulatory Pediatric Association, American Academy of Pediatrics, American Association of Colleges of Nursing, Association of Academic Health Center, and National Association of Pediatric Nurse Practitioners.

The planning committee in consultation with their respective professional organizations and colleagues identified faculty members who were interested in becoming PEH faculty champions at their academic institutions [National Environmental Education and Training Foundation (NEETF) 2002]. These faculty champions agreed to take a leadership role in integrating PEH into their institutions in a sustainable fashion, lend expertise and support in their institutions and surrounding communities, teach courses, integrate competencies into curriculum, and serve as a model for how to integrate environmental health into health professional education. Although participants were selected from a wide geographic distribution, the convenience sample included 28 faculty members from U.S. medical, nursing, and physician assistant schools: 15 physicians (1 department chair, 1 section chief, 1 director of pediatric residency program, and 13 associate or assistant professors), 7 nurse practitioners (2 program directors and 5 clinical professors), 5 nurses with graduate degrees (2 associate deans, 1 director, 1 assistant director, and 1 project director), and 1 dean of physician assistant studies with a doctoral degree. There were 15 males and 13 females in the sample. Ethnicity, race, and age data were not collected. For the purpose of cross-fertilization between medical and nursing professionals, we drew faculty from academic health centers with both a medical and nursing school. A special emphasis was placed on including faculty members who served minorities and underserved communities. To this end, participants included faculty from historically black colleges and universities and a representative from National Medical Association.

### Data collection

#### Train-the-Trainer Workshop

The 1-day train-the-trainer workshop was held in 2006, and participants received continuing education units and continuing medical education credits for the workshop. Six instructors experienced in PEH led the workshop and compiled the curriculum from NEEF’s peer-reviewed children’s environmental health medical and nursing training materials, the medical/health literature, and materials from the Agency for Toxic Substances and Disease Registry and Association of Occupational and Environmental Clinics’ resources.

Instructors presented material on six topic areas: environmental history taking; environmental management of pediatric asthma; environmental tobacco smoke; ultraviolet light; pesticides; and lead and mercury. After each topic was presented, an opportunity for discussion of questions and comments was provided. In breakout sessions participants discussed strategies for integrating environmental health into education and practice as well as training other faculty members. Faculty champions also developed individual action plans describing opportunities, barriers, strategies, and planned activities for both training faculty and students and integrating environmental health into practice and curricula. A reader analyzed all qualitative data by reviewing for common themes, and a second reader validated the findings. Workshop participants evaluated each of the six topic presentations on a 4-point scale: 1 = highly effective, 2 = moderately effective, 3 = somewhat effective, and 4 = not effective. The evaluation included a section for comments. NEEF provided faculty champions with several tools to be used for future lectures, workshops, or training sessions. These included PowerPoint presentations on each workshop topic and NEEF’s peer-reviewed resources such as pediatric environmental history forms and *Environmental Management of Pediatric Asthma: Guidelines for Health Care Providers* (NEETF 2005). Throughout the project period, NEEF provided ongoing support to the faculty champions in several ways, including creating a listserv to facilitate communication and networking among the faculty champions, informing faculty champions of PEH literature and training opportunities, providing planning and implementation support, and providing materials for use and distribution at their trainings.

### Pretest/posttest

Investigators developed a pretest and posttest tool from questions and answers submitted by workshop instructors based on the six topic areas presented at the workshop and reflecting the competencies, for a total of 20 questions. Five health professionals pilot-tested the pretest for content validity, and the pretest was revised and distributed to all workshop participants via email 1 week before the workshop. Faculty champions completed the first posttest at the conclusion of the workshop to measure knowledge gained, and completed the second posttest 3 months after the workshop to measure knowledge sustainment. Pre- and posttest data were evaluated using two-way analysis of variance (ANOVA).

### Baseline data and progress assessments

Investigators developed two progress assessment rating scale forms, baseline and ongoing, to determine the extent to which PEH competencies were taught as part of curriculum, the faculty champion’s personal use of these competencies in practice, and the degree of competency achievement. Each faculty champion indicated self-assessment and curricula assessment on each competency as 0 = not applicable, 1 = not done, 2 = to little extent, 3 = to moderate extent, and 4 = to great extent. Competency assessments were then averaged. The progress assessment form included several additional questions, including the number of faculty members trained on PEH topics and number of PEH referrals made. The form included a section to provide two to three examples of how faculty champions incorporated PEH into curricula and practice. NEEF staff electronically mailed forms to participants, who then returned the forms electronically or by fax. Faculty champions completed the initial baseline assessment form within 1 month after the workshop and completed ongoing progress assessment forms 4 and 8 months after the workshop.

### Telephone interviews

Investigators developed an assessment form for telephone interviews to obtain direct information about methods used for incorporation of PEH into curricula and practice, including the development of any institutional intervention and materials, such as policies and protocols, as well as feedback and suggestions for program improvement. A trained research assistant and NEEF staff conducted interviews at 6 and 12 months after the workshop. Interviewers asked faculty champions about the sustained use and degree of incorporation of PEH history taking into curricula, individual practice, and institutional practice protocols, procedures, and policies. Interviewers queried faculty champions’ methods of integration, topics covered, and time committed to integration approaches. The faculty champions also provided two or three examples of strategies used in practice that resulted in specific behavioral changes in parents and children, as well as institutional changes. The interviewer handwrote responses and later transcribed them into a typed document. During the telephone interview at 12 months, participants reviewed their responses from the interview at 6 months. The interviewers asked participants for updates on their progress and asked about future plans to sustain efforts regarding PEH inclusion in curricula and practice.

## Results

### Workshop evaluation

Participants (*n* = 25) who completed the workshop evaluation rated the content highly effective, with an average score for all topic areas as 1.35. Average scores for individual topic areas ranged from 1.16 to 1.48. Participants’ comments were highly favorable.

### Action plan

During the workshop, 27 of the 28 faculty champion participants completed an action plan to identify opportunities, barriers, strategies, and planned activities for training faculty members and integrating environmental health into education and practice. The common themes identified are shown in Appendices 1 and 2.

### Pretest/posttest

Faculty champions completed a pretest and two posttests related to the workshop content on environmental health. A total of 82 tests (28 pretests, 28 first posttests, and 26 second posttests) were completed for analysis evaluating the difference in means between the examinations using two-way ANOVA. Improvement was seen in the faculty champions’ scores between each test. The mean score of 52% achieved at the pretest was increased by 13.5% at the first posttest, immediately after the workshop, for a mean score of 65.5% (*p* = < 0.0001). Concurrently, the average percentage correct for each of the 20 individual questions increased by 13.3% from 52% to 65.3%. For the first posttest, 10 participants improved their scores 20% by correctly answering at least 4 additional post-test questions, and 14 participants answered from 1 to 3 additional posttest questions correctly. Average scores for the second post-test, completed by 26 participants 3 months after the workshop, increased significantly by 19.5% (52% to 71.5%, *p* = < 0.0001) from the pretest and 6% (65.5% to 71.5%, *p* = 0.2266) from the first posttest, showing sustained knowledge. Of the participants who completed the second posttest, 14 (54%) equaled or improved their scores between the first and second posttests and participants answered > 80 questions correctly from the pretest responses.

### Baseline data and progress assessments

Faculty champions provided baseline data within 1 month of the workshop and ongoing data at 4 (time 1) and 8 (time 2) months after the workshop. Rating scores for each of the five competencies were averaged for each participant on both professional practice self-assessments and curricula assessment, giving a composite rating for each participant and time interval. All competency areas combined showed improvement except for reporting exposure incidents in the practice setting, as seen in Table 1. Faculty champions reported integration of PEH content into curricula and practice in several ways as shown in Appendix 3.

**Figure 1:**
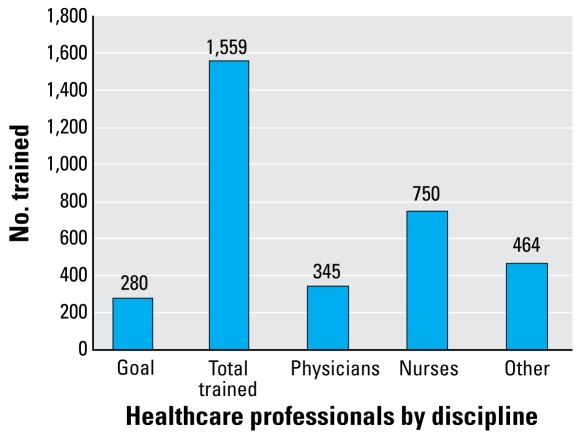
Health care professionals trained by faculty champions within 12 months of the train-the-trainer workshop, by total number and discipline.

Additionally, the faculty champions reported on the number of health professionals they trained within the project period. The project goal was set at 280 health providers trained, or 10 trainees per each of the 28 faculty champions. As shown in Figure 1, 1,559 health professionals were trained in the 12 months after the workshop: 345 physicians, 750 nurses, and 464 others (primarily medical students, residents, and physician assistants). The faculty champions were successful in training their colleagues and community members, substantially exceeding the goal of 280 trainees, with a rate of 55.7 trainees per faculty champion or a total rate of 5.6 over the expected 280 trainees. Furthermore, through the faculty champions’ involvement in other activities, such as presenting at national conferences and publishing in journals, additional health care professionals were exposed to PEH information.

### Telephone interviews

Faculty champions were contacted via telephone interview at 6 and 12 months after the training workshop. Twenty-four of 28 faculty members completed the first telephone interview. Of the faculty champions who participated in the telephone survey, 87% reported PEH integration into the curricula at their respective institutions. Activities for curricula integration ranged from presenting environmental health topics at lectures, grand rounds, noon conferences, and national conferences of health professional organizations to more sustainable efforts, such as developing online courses and modules for PEH topics, integrating PEH topics into required courses, creating a center for environmental health, and forming a PEH residency certification program in which residents complete 18 PEH modules. New integrated content focused on taking an environmental history, targeting toxic exposures, and evaluating findings. Only 16.7% participants reported formal policy changes in their institutions. The amount of time committed to PEH content was varied, ranging from 0.5 to 20 hr/year. In the second telephone interview, 20 of 28 faculty champions participated and reported building on the activities mentioned in the first interview and continuing to add PEH content into lectures and courses. Results were slightly higher in the second interview, with 90% of participants reporting PEH integration into curricula and 26.3% reporting institutional policy changes.

Faculty champions provided examples of additional PEH activities. These included working with migrant workers and their families, teaching high school students interested in health careers about PEH, and working with state officials to make environmental health policy recommendations. The second telephone interview included a request for feedback on the program. Many participants indicated that they found the overall program valuable. Participants reported feeling more confident about their knowledge of environmental health issues and ability to teach the material more effectively.

Faculty champions indicated that the overall impact of the training was enormous and resulted in sustained changes in curriculum and changes in students’ regard of PEH integration into their learning. Faculty champions’ recommendations for the program included clustering participants so that several faculty champions would come from each institution, forming a critical mass; bringing the faculty champions together in the months after the workshop to discuss successful strategies for integrating environmental health education into each institution; and inviting more physician assistants to participate in the program. Faculty champions also indicated they would continue their PEH efforts even after the project period ended. Faculty champions reported an interest in participating in future training to share lessons learned from their experiences and serve as mentors for a new group of faculty champions.

## Discussion

The Children’s Environmental Health Faculty Champion Initiative was successful in building capacity among health care providers in PEH. The initiative resulted in increased PEH knowledge among faculty champions, the education of a significant number of health care providers in PEH, and sustained changes in knowledge, practice, curricula, and institutional policies.

The train-the-trainer workshop attended by the faculty champions was an effective strategy for initiating the program, educating participants, providing tools and resources to faculty champions, and developing individual action plans to achieve the program goals. In the workshop evaluation, the sessions were rated highly effective by the participants, many of whom have had little previous exposure to the content provided. The workshop was highly successful in significantly increasing faculty champions’ knowledge of PEH issues, which was sustained over at least a 3-month period. The significant change in pre- and posttest scores provided objective evidence of the limited knowledge health care professionals began with in PEH and supports the contention of several investigators that environmental health content is lacking in medical and nursing school curricula (Balbus et al. 2006; Kilpatrick et al. 2002; McCurdy et al. 2004). An increase in knowledge is the essential initial step in incorporating PEH information into health professionals’ education and practice.

Progress assessments indicated that the areas in which participants reported being the most competent were taking a PEH history and resource use for environmental health hazards. These topics are more basic components of PEH and were a main focus of the workshop. This result is particularly important and demonstrates the potential impact of this workshop, as previous studies have found a lack of consistent and comprehensive environmental health history-taking (Balbus et al. 2006; Kilpatrick et al. 2002; Woolf and Cimino 2001).

Areas in which participants reported being moderately competent were making referrals and involvement with the community. Participants reported being the least competent in reporting incidents in order to comply with regulatory requirements. To properly submit a report of an incident-related exposure, one must first detect an environmental toxicant-related disease. Regulatory-required reporting usually follows accurately detecting environmental toxicant–related disease—a complex skill. The complexity involved may explain the decreased comfort of practitioners in these areas. Also, there could have been uncertainty about what to report or where to report, or that specific incidents did not present themselves. It is also possible that respondents forgot some of the incidents they reported, or did not want to get involved with regulatory issues. Consequently, this may suggest an increased need for training regarding regulatory requirements and reporting incidents.

Review of the action plans completed at the workshop resulted in seven themes for training faculty members and eight themes for practice and education integration. Except for one action plan strategy—discussion with certification boards about incorporating PEH content questions on examinations—all strategies were achieved, demonstrating the remarkable effort and commitment by the faculty champions in achieving their goals.

Over a period of 12 months, faculty champions reported continuing efforts to integrate PEH into curricula and implement policy changes at their respective institutions. Faculty champions also reported changes to protocols and procedures, including the adoption of environmental health history–taking forms at their institutions and instituting chart audits to evaluate whether PEH histories were being performed. Five respondents reported changes related to smoking cessation, including the development of a smoking cessation clinic, second hand smoke training, and audits to ensure that environmental tobacco smoke exposure screenings were being performed. Telephone interviews conducted at 6 months and validated at 12 months showed sustained and increasing integration of PEH content into practice and policy changes over the 1-year period of time. Faculty champions indicated that the overall impact of the training was very positive considering the difficult task of participants to spread knowledge to a larger audience, begin to influence institutional and curricula changes, distribute educational materials to faculty and students, champion changes regarding integration of pediatric and environmental health emphasis into students’ learning, discuss with the community and policy makers about environmental health issues, and increase personal knowledge about PEH.

Because this original study used a pre-experimental design, some limitations exist. Although the sample size of 28 was small, this was consistent with the design plan. In addition, faculty were volunteers who were hand-picked based on their interest in the topic content. However, efforts were made to have representation from the various disciplines in academic medical centers. This does limit the ability to generalize the findings to this group. The lack of a comparison group prevents the ability to determine that the independent variable (the NEEF workshop) affected the dependent variables (knowledge gained, changes in practice, and incorporation of PEH into curricula). The lack of a comparison group also brings the potential for threats to internal validity, such as attrition, history, and maturation. Because the study lasted > 1 year, these threats are amplified, and also may have contributed to fatigue of participants over time. Lack of institutional support is another limitation. Nevertheless, because this project was conducted in a real-world setting with limited resources, it is inevitable that some limitations exist.

Studies support that faculty leadership is key to integrating prevention-related topics (Lindberg 1998; Sachdeva 2000; Skochelak et al. 2001; Susman and Pascoe 2001) into curricula, with the creation of faculty leaders as one of the key methods to build environmental health capacity among health care professionals (McCurdy et al. 2004). Investigators have reported that faculty members can use their leadership role to implement curricula, influence the career choices of students, introduce topics that serve as an impetus for change, advocate for research funding, and ensure that the content material is taught in their classes (Goldman et al. 1999; Schwartz et al. 1995).

Studies have shown that primary-care residency faculty trained in environmental/occupational health have increased the environmental/occupational health education offered at their schools, and after physicians attended an interactive asthma seminar, the children they saw experienced fewer hospitalizations and fewer subsequent emergency department visits (Clark et al. 2000; Frazier et al. 1999). Another study that evaluated pediatric primary care providers’ opinions on continuing medical education showed that in-person training sessions, especially lectures and short courses, were the modes of education pediatricians and nurses preferred most (Balbus et al. 2006). In this study, faculty champions trained > 1,500 health care professionals and integrated PEH into curricula, thereby demonstrating a successful model for increasing the cadre of practitioners and educators who are more competent in PEH.

Faculty champions faced numerous barriers to information integration: time limitations in practice settings, competing time constraints in a busy academic career, a perceived or actual lack of influence in their institutional and practice settings, and inability to change institutional and practice settings (i.e., lack of support and knowledge of PEH by upper management). Many faculty champions stated that change in academic settings is a slow and difficult process. Finally, faculty champions were unable to follow up with their trainees to evaluate gains in knowledge and behavior changed. This was possibly attributable to the faculty champions’ busy schedules and the lack of a grant or stipend so they could justify their time spent on the project.

As part of the overall initiative, NEEF provided support to the faculty champions and conducted outreach throughout the project period. One of the institutional changes achieved through this comprehensive approach was the inclusion of the PEH history forms in *Pediatric Primary Care* (Burns et al. 2008). This textbook is used in educational institutions that train pediatric nurse practitioners, family nurse practitioners, physicians, and physician assistants. This inclusion advances the integration of pediatric environmental history taking in curricula and practice.

Future programs could include training on instituting policy changes and involvement of at least two representatives from each institution for increased faculty champion support. Furthermore, the use of teleconferences and webinars could be helpful in supplementing information, promoting knowledge sustainment, and supporting continued competency achievement. This is consistent with the findings of D’Eon and AuYeung (2001) who reported that audio teleconferences following train-the-trainer programs provided the opportunity to engage in professional discussion and aided in practice changes.

Future research should consider whether program content should vary among practitioner types (i.e., nurse, nurse practitioner, physician, physician assistant), and which teaching and outreach strategies work best. Methods to effect policy change in practice and educational institutions and approaches needed to sustain knowledge and practice integration should be evaluated. In addition, a pilot study on a larger sample size of participants who could be drawn from clustered groups of schools of nursing, medicine, or physician assistants might be considered. Furthermore, a systemic comprehensive study of similar training programs would be valuable for the field.

## Conclusion

There is a need for environmental health education in pediatric medicine and nursing. NEEF’s Children’s Environmental Health Faculty Champions Initiative was a highly effective method of building environmental health capacity among health care professionals. Faculty champions made significant progress in integrating environmental education into the curricula and practice at their institutions, and exceeded their target number of 280 health care professionals by training 1,559 trainees through lectures, presentations, grand rounds, faculty discussions, and noon conferences. This is a replicable method that can be modeled in other arenas.

## Figures and Tables

**Table 1:** Faculty champions’ composite scores: competency data assessments.

	Assessment interval					
	Integration into practice			Integration into curriculum		
Competency	Baseline	Time 1	Time 2	Baseline	Time 1	Time 2
PEH history taking	2.4	2.7	3.1	2.2	2.5	2.9
Making referrals	2.2	2.3	2.5	2.0	1.9	2.1
Involvement with community groups/organizations	2.0	2.3	2.3	1.7	2.0	2.3
Use of resources	2.5	2.7	2.9	2.1	2.2	2.7
Reporting incidents	2.2	1.6	1.6	1.7	1.4	1.8

Scale: 1 = not done, 2 = to little extent, 3 = to moderate extent, 4 = to great extent. Baseline Assessment = 1 month after training workshop. Time 1 = 4 months after training workshop. Time 2 = 8 months after training workshop.

## Appendix 1 Action plan responses with common themes of training faculty members

- Discuss course work in environmental health content in nursing, medical, pediatric, and physician assistant courses with faculty and course directors and add content appropriate to undergraduate and graduate-level courses, online/web-based courses, and continuing education courses
- Presentcontent at local and national conferences
- Discuss with certification examination boards the inclusion of environmental content questions
- Present environmental health content information at grand rounds, brown bag discussions, and faculty meetings
- Discuss possibility for research opportunities in environmental health
- Talk with community providers/policy makers to include environmental health content as outreach activities and in school-based programs
- Revise assessment/screening template and clinic/patient care forms to include environmental health questions

## Appendix 2 Action plan responses with common themes of integrating PEH into education and practice

- Reinforce with faculty to include environmental health content and provide content resources, website information, and web lists
- Encourage environmental health clinical rotations, presentations at noon conferences for residents/staff, and discussion and feedback
- Give presentations at conferences and find opportunities for networking with other faculty champions
- Contact certification boards for environmental health content addition
- Encourage faculty champions to act as role models for environmental health integration
- Revise records/forms to include environmental health
- Meet with community advocacy groups, public health nurses, and parents to discuss environmental health issues
- Foster environmental health research

## Appendix 3 Examples of PEH content integration into curricula and practice

- Added lecture content to graduate and undergraduate nursing courses and to medical school courses
- Increased emphasis on PEH history taking
- Changed assessment forms in patient records to include questions on environmental health hazards
- Published in journals on environmental health
- Provided clinical rotations related to environmental health
- Held noon conferences on environmental health topics
- Created smoking cessation programs
- Initiated chart audits for environmental health
- Performed grand rounds on environmental health
- Increased awareness by residents and nursing students to ask about environmental health issues
- Presented at National Nursing Conference
- Presented at four physician assistant conferences
- Presented training sessions for nurses, nursing students, medical students, residents, and physicians
- Added information to websites and Youtube
- Presented content on environmental health risks in developing countries, Hispanic initiative, and others
- Increased exposure in clinical rotations and increased counseling on PEH topics with patients
- Increased emphasis on lead and mercury screening and health assessments on environmental toxins to physicians, nurses, and for students
- Added PEH content in coursework and noon conferences for physicians
- Participated in research studies with environmental health emphasis
- Developed online courses and modules
- Added advocacy rotation in residency program involving home lead inspections
- Implemented PEH residency certification program
- Created center of environmental health at institution
- Provide PEH lecture and discussion during required clerkship
